# Advances in the Pathogenesis of Cardiorenal Syndrome Type 3

**DOI:** 10.1155/2015/148082

**Published:** 2015-03-04

**Authors:** Anna Clementi, Grazia Maria Virzì, Alessandra Brocca, Massimo de Cal, Silvia Pastori, Maurizio Clementi, Antonio Granata, Giorgio Vescovo, Claudio Ronco

**Affiliations:** ^1^Department of Nephrology and Dialysis, San Giovanni Di Dio, Agrigento 92100, Italy; ^2^Department of Nephrology, Dialysis and Transplant, San Bortolo Hospital, Vicenza 36100, Italy; ^3^International Renal Research Institute Vicenza (IRRIV), Italy; ^4^Clinical Genetics Unit, Department of Women's and Children's Health, University of Padua, Italy; ^5^Department of Medicine (DIMED), University of Padova, Padova 35128, Italy; ^6^Internal Medicine, San Bortolo Hospital, Vicenza 36100, Italy

## Abstract

Cardiorenal syndrome (CRS) type 3 is a subclassification of the CRS whereby an episode of acute kidney injury (AKI) leads to the development of acute cardiac injury or dysfunction. In general, there is limited understanding of the pathophysiologic mechanisms involved in CRS type 3. An episode of AKI may have effects that depend on the severity and duration of AKI and that both directly and indirectly predispose to an acute cardiac event. Experimental data suggest that cardiac dysfunction may be related to immune system activation, inflammatory mediators release, oxidative stress, and cellular apoptosis which are well documented in the setting of AKI. Moreover, significant derangements, such as fluid and electrolyte imbalance, metabolic acidosis, and uremia, which are typical features of acute kidney injury, may impair cardiac function. In this review, we will focus on multiple factors possibly involved in the pathogenesis issues regarding CRS type 3.

## 1. Introduction

Heart performance and kidney function are closely interconnected. The physiological crosstalk between these two organs is necessary to maintain the regular homeostasis and the normal functioning of the human body. However, during disease states, the damaged organ can induce structural and functional dysfunction in the other organ [1]. Acute and chronic cardiac disease, indeed, can directly contribute to concurrent acute and chronic worsening of kidney function and vice versa [2, 3]. The term cardiorenal syndrome (CRS) is often used to describe these clinical conditions [2, 4]. The classification into five subtypes is based on the primary organ dysfunction, whether heart or kidney, and on whether the organ dysfunction is acute or chronic [2] (Table 1). It is known that many patients may transition between different CRS subtypes during the course of their disease [5].

In the critical care setting, acute kidney injury (AKI) remains an important predictor of clinical outcome and frequently results in remote organ dysfunction involving the heart, lung, liver, intestines, and brain through immune mediated inflammatory mechanisms [6–8]. Moreover, significant derangements, such as fluid and electrolyte imbalance, metabolic acidosis, and uremia, which are typical features of AKI, may be responsible for distant organ impairment. In this review, we will focus on multiple factors possibly involved in the pathogenesis issues regarding CRS type 3.

## 2. Pathogenesis

The pathophysiologic mechanisms underlying the interplay between kidney injury and heart dysfunction are still not completely understood. It is well known that a decline in cardiac function causes a decrease in tissue perfusion and thus adversely affects renal perfusion leading to renal injury. On the other hand, AKI can affect the heart through several pathways which can be divided into two categories: the* direct effects* of AKI on heart and the effects of AKI on remote organ function with* indirect effects* on the heart (Table 2) [9].

**Figure 1:**
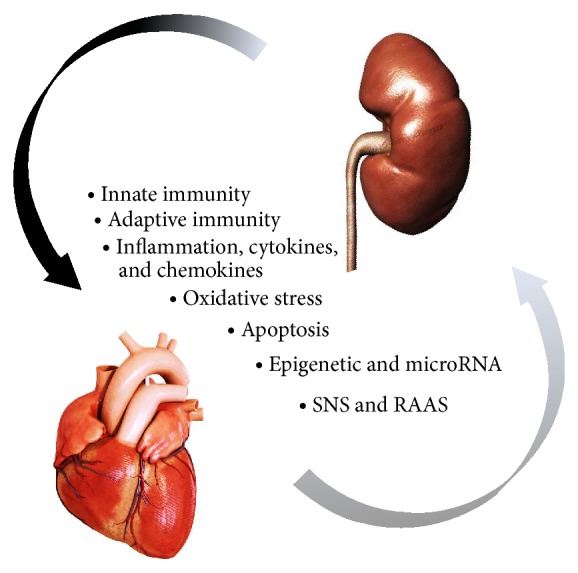
Direct mechanism of cardiorenal syndrome type 3.

The direct physiologic mechanisms by which AKI interacts with the heart have been defined as “cardiorenal connectors” [10] and they involve the activation of the systemic immune system, inflammatory mediators release, oxidative stress, cellular apoptosis, and the activation of the sympathetic nervous system (SNS) and the renin-angiotensin-aldosterone system (RAAS) (Figure 1). Fluid overload, electrolyte imbalances, acidemia, and acute accumulation of uremic toxins are the most important indirect mechanisms involved in the pathogenesis of CRS type 3.

## 3. Direct Effects

### 3.1. Innate and Adaptive Immunity

The immune system is divided into the innate or nonspecific immune system and the adaptive or specific immune system. Recent studies have highlighted the importance of both immune responses to endogenous molecules induced by either tissue damage or infection [11, 12]. The innate immune system is our first line of defense against invading organisms. It is immediately activated in infection states and inflammatory conditions in a nonantigenic-specific way. The adaptive immune system acts as a second line of defense and also affords protection against reexposure to the same pathogen. Dendritic cells and macrophages play important functions in both types of immunity by generating cytokines and chemokines and presenting antigens to lymphocytes [11, 13, 14].

The majority of experimental studies evaluating the immune “cardiorenal connectors” have focused on renal ischemia and ischemia/reperfusion injury (IRI). In IRI models, AKI has been shown to elicit a systemic immune response characterized by dose-response increase in circulating levels of pro- and anti-inflammatory mediators. Innate leukocytes, endothelial cells, and epithelial cells contribute to early ischemia-reperfusion injury with subsequent inflammation [13, 15–17]. In response to hypoxia-reoxygenation, early immune cell infiltration seems, indeed, to activate resident kidney dendritic cells, which are localized in the interstitial extracellular compartment throughout the whole organ [18, 19]. The early immune response consists of activation of dendritic cells and dual activation of interleukin (IL-) 12/interferon-*γ* (IFN-*γ*) and IL-23/IL-17 signaling pathways [17, 20, 21]. Macrophages, neutrophils, and lymphocytes, particularly CD4^+^ T cells and B cells, are thought to contribute to kidney IRI [13, 21–23]. Antigen-dependent T cell activation has been demonstrated in experimental models of renal IRI as well [24, 25]. Either following antigen activation or in the presence of chemokines, T cells undergo early activation as a bridge between adaptive and innate immune systems. This specific immune response in the setting of AKI enhances the heart-kidney crosstalk [14].

AKI seems to induce functional alterations in immune cell responsiveness and in leukocyte trafficking, adhesion, and tissue extravasation both locally in the kidney and in distal organs, such as in the heart [6, 14, 16, 26]. Neutrophils, macrophages, natural killers, and lymphocytes infiltrate into the injured kidneys. The injury prompts the activation of inflammatory pathways by tubular and endothelial cells recruiting leukocytes into the kidneys [13, 27, 28].

Adherence of neutrophils to the vascular endothelium is a crucial early process in the initiation of damage to ischemic tissues. One of the hallmarks of renal IRI, in mouse models, is neutrophil accumulation in the postischemic kidney and depletion of neutrophils preventing AKI [29–31]. During AKI, chemokines recruit inflammatory cells with a consecutive neutrophil infiltration into the heart tissue and is a causal factor of myocyte apoptosis [13].

After adherence and chemotaxis, neutrophils release reactive oxygen species, proteases, myeloperoxidase, and other substances that directly induce tissue damage or stimulate the expression of cytokines which play a pivotal role in heart failure [32, 33]. Cardiac immune cell infiltration contributes to cellular proliferation and inflammation, thus inducing myocardial hypertrophy and fibrosis.

Moreover, elevated white blood cell count has been found to increase the risk of acute myocardial infarction [34–37].

Burchill et al. observed that, in a rat model, kidney injury of a short duration of 10 days resulted in cardiac remodelling, as evidenced by hypertrophy and by interstitial and perivascular fibrosis, and that these changes were associated with increased expression of ACE2. These data suggested that AKI promoted adverse cardiac remodelling with cardiac fibrosis and hypertrophy as early as 10 days after injury [38].

Furthermore, Bozkurt et al. underlined that, in rat model, pathophysiologically relevant concentrations of tumor necrosis factor-*α* (TNF-*α*) exerted profound effects on left ventricular remodelling, myocyte hypertrophy, and apoptosis, ultimately resulting in a progressive reduction of myocardial contractility [39].

### 3.2. Inflammatory Mediators and Oxidative Stress

Inflammation is a pathophysiological response to infection or tissue damage. Initially, tissue-resident cells of the innate immune system detect the damaging insult and alarm circulating neutrophils. These migrate to the inflamed tissue, promote recruitment of inflammatory monocytes, and potentiate the proinflammatory environment [40]. Under normal conditions, neutrophils undergo apoptosis after performing their action at the inflamed site [41] and macrophages ingest apoptotic neutrophils. Clearance of apoptotic neutrophils prompts a switch from a pro- to an anti-inflammatory macrophage phenotype [42], which is a prerequisite for macrophage egress via the lymphatic vessels, thus permitting tissue homeostasis to be maintained [43].

Nonresolving and persistent exposure to proinflammatory factors impairs organ function. Several studies have found circulating levels of TNF-*α*, IL-1, and IL-6 to increase immediately after renal IRI in experimental models [28, 39, 44–47]. Moreover, 48 hours after kidney ischemia-reperfusion injury, hearts seem to develop functional changes, such as left ventricular dilation, increased left ventricular end diastolic and end systolic diameters, increased relaxation time, and decreased fractional shortening. These cardiac changes are associated with the increase in TNF-*α*, IL-1, IL-6, and intercellular adhesion molecule (ICAM)-1 [6, 10, 28, 48]. Interestingly, these heart modifications have not been found in case of bilateral nephrectomy, suggesting that IRI and not uremia per se contributes to this effect [49].

Moreover, accumulating evidence from various animal studies has supported the systemic protective potential offered by remote ischemic preconditioning (RIPC) on heart and kidney function [50, 51]. Ischemic preconditioning is an adaptive response of briefly ischemic tissues which serves to protect against subsequent prolonged ischemic insults and reperfusion injury. The RIPC stimulus presumably induces the release of biochemical messengers which reduce oxidative stress and preserve mitochondrial function. Several studies showed that brief renal ischemia reduced the size of infarct resulting from myocardial ischemia [52–54]. The onset of myocyte injury is associated with adenosine triphosphate (ATP) depletion and breaks in sarcolemmas, responsible for intracellular acidosis. Takaoka et al. showed that renal artery occlusion (10 minutes) followed by reperfusion (20 minutes) in rabbits led to a decrease in myocardial infarct size on histology and attenuated ATP depletion and preservation of myocyte pH during subsequent ischemia-reperfusion [53]. Further studies are needed to evaluate the possible role of remote ischemic preconditioning in the pathogenesis of CRS type 3.

Given the immune activation occurring in case of heart failure, adhesion molecules may play a critical role in both local and systemic inflammatory responses. In fact, soluble ICAM-1 has been shown to be upregulated in patients with chronic heart failure and a significant negative correlation between left ventricular ejection fraction and soluble ICAM-1 levels has been observed [55].

During AKI, there is an increased amount of both cardiac and systemic TNF and IL-1 along with an increased expression of ICAM-1 messenger RNA, selectin, complement activation, reactive oxygen species, nuclear factor-kB activation, and toll-like receptor-related pathway. Decreased renal ischemia time seems to attenuate cardiac apoptosis and to reduce IL-1 and ICAM-1 levels [9]. However, other mechanisms may contribute to the increased levels of serum cytokines independent of the production by the kidney. In AKI, cytokines may be produced by other tissues and cleared by the kidney [56].

Although proinflammatory cytokines exert negative inotropic effects, the nature of the inotropic response is more complex and dependant on the production of secondary mediators as well [44]. Nitric oxide (NO) derived from constitutive NO synthase, sphingolipid mediators, and arachidonic acid have been shown to contribute to the immediate contractile effects, while the delayed response results primarily from the combined NO, the production of radical oxygen species, and alterations in *β*-adrenergic receptor signaling [44].

### 3.3. Apoptosis

Apoptosis is an active mode of cellular death necessary for normal cellular development, aging, and tissue homeostasis [57]. It is initiated by the activation of cell death receptors and in most cases it is associated with the activation of the cysteine proteases (caspases), which inactivate various structural and functional intracellular proteins essential for cell survival and proliferation. An alteration in the regulation of cell death by apoptosis may negatively affect the mechanism of host defense [57]. Cardiac myocyte apoptosis and neutrophil infiltration are two of the most important contributors to the pathophysiology of myocardial infarction during AKI and transgenic rat models have shown that even apoptosis can lead to tissue damage and lethal heart dysfunction [28]. In particular, Kelly demonstrated that kidney IRI but not uremia is fundamental to trigger apoptosis in myocardial tissue [28]. Another experimental rat model of cisplatin-induced AKI found a significantly increased level of myocardial apoptosis by TUNEL assay [58]. Moreover, TNF-*α* contributes directly to cardiomyocyte apoptosis, as demonstrated by attenuation of apoptosis following administration of anti-TNF-*α* antibodies [28].

### 3.4. SNS and RAAS

There is limited data describing the role of the SNS and RAAS in the pathogenesis of CRS type 3. Sympathetic activation influences intrarenal hemodynamics and stimulates renin secretion by the juxtaglomerular apparatus of the kidney. This initial activation of the SNS is a protective mechanism able to maintain cardiac output. Unfortunately, it may impair myocardial function through several mechanisms, such as direct effects of norepinephrine, disturbances in myocardial calcium homeostasis, increase in myocardial oxygen demand, cardiac myocyte apoptosis mediated through *β*_1_-adrenergic receptors stimulation, and direct activation of RAAS [59–62]. Moreover, SNS activation seems to increase neuropeptide Y, a vascular growth-promoter, responsible for neointimal formation, vasoconstriction, and impairment of immune system function [63].

RAAS stimulates sodium reabsorption in the proximal tubule and constriction of the efferent arteriole, thus leading to increased intraglomerular capillary pressure and filtration fraction, able to maintain glomerular filtration despite a decreased renal blood flow. Unfortunately, inappropriate RAAS activation in AKI contributes to angiotensin II release, vasoconstriction, and further loss of extracellular fluid homeostasis. Angiotensin II may also play a direct role in modifying myocardial structure and function [64], contribute to cellular hypertrophy, and precipitate apoptosis in cardiac myocyte cultures [65]. It is also responsible for the activation of several cell signaling pathways including oxidative stress, inflammatory mediators release, and extracellular matrix regulation [66]. Angiotensin II can indeed activate the enzyme NADPH oxidase in endothelial cells [67, 68], vascular smooth cells, renal tubular cells [69], and cardiomyocytes [70] and may lead to the formation of reactive oxygen species [71].

### 3.5. Epigenetics and microRNA

Epigenetics plays a crucial role in mammalian development and in several pathological conditions, such as cancer and immune dysfunction. Epigenetic mechanisms include DNA methylation, histone modification, and RNA interference and are associated with chromatin remodeling and gene expression regulation. Bomsztyk and Denisenko have provided an overview of the mechanisms underlying cellular response to AKI as well as growing evidence that epigenetic factors are involved in the regulation of genes associated with AKI [72]. It is known that DNA methylation and histone modifications closely interact to control gene expression [73] and they may have a role in the organ-crosstalk [74]. Epigenetics inheritance is responsible for a huge number of phenotypic differences between cells in multicellular organisms [75]. This may explain why subjects having similar genetic background and both environment and classical risk factors for cardiovascular disease and/or chronic kidney disease could have a very different outcome in clinical manifestation of these diseases [74]. Furthermore, microRNAs (endogenous noncoding RNAs (~22 nucleotides) that regulate gene expression at the posttranscriptional level) are important for kidney and heart development and homeostasis and are known to play a pathogenic role in cardiac and renal diseases [74]. In particular, microRNAs have been implicated in cardiac remodeling and regeneration [76]. Limited data are available about epigenetics mechanisms and cellular responses associated with CRS Type 3 and in general with cardiorenal syndrome. Recently, evidence is emerging about overnutrition and CRS. Nistala et al. reported that overnutrition in both mothers and fathers may affect fetal programming and may predispose the fetus and future adult to CRS [77]. However, it is still unclear how CRS risk factors are affected by histone modification, methylation, and RNA interference. Furthermore, it would be noteworthy to understand whether drugs and therapies modulating epigenetics modifications could prevent CRS. Thus, a better understanding of epigenetics mechanisms in cardiorenal context could revolutionize both diagnosis and treatment of CRS.

## 4. Indirect Mechanisms

It is well known that AKI results in significant physiological derangement that may lead to cardiac injury.

### 4.1. Fluid Overload

Oliguria can lead to fluid overload and sodium and water retention, contributing to the development of systemic edema, cardiac overload, hypertension, pulmonary edema, and myocardial dysfunction [78]. Excess fluid accumulation seems to be associated with less favorable outcomes in critically ill patients [59, 79]. Fluid overload may indeed worsen kidney function through venous congestion and intra-abdominal hypertension. Moreover, fluid removal in critically ill patients, either with loop diuretics use [80] or ultrafiltration [81], has been demonstrated to increase patients' survival.

### 4.2. Electrolyte Imbalances

Hyperkalemia, may be responsible for an increased risk of arrhythmias and even cardiac arrest. Hyperphosphatemia also can cause arrhythmias and depress myocardial contractility. While untreated AKI is often associated with elevated plasma phosphate levels, patients started with renal replacement therapy (RRT) commonly develop hypophosphatemia [82–84]. Hypophosphatemia may be unrecognized iatrogenic complication associated with respiratory muscle weakness and impaired myocardial performance [85, 86]. Calcium plays a central role in the regulation of myocardial contraction and relaxation and there is increasing evidence that disturbances in calcium handling may disturb contractile cardiac function [59, 87–89]. Severe* hypermagnesemia* can cause atrioventricula (AV) nodal and intraventricular conduction disturbances that may culminate in complete heart block and cardiac arrest [59, 90].

### 4.3. Acidemia

It appears to disturb cardiac energetic metabolism and to induce pulmonary vasoconstriction, thus increasing right ventricular afterload. The accumulated acid (H^+^) may alter protein structure and interfere with its normal function. This may directly contribute to decreased myocardial contractility through changes in *β*-receptor expression and altered intracellular calcium handling [89].

### 4.4. Uremic Toxins

In chronic kidney disease (CKD), more than 100 uremic toxins or retention products have been identified [91]. Acute accumulation of uremic toxins, including the nitric oxide synthase-modulating guanidine-succinic acid, and methylguanidine may lead to myocardial ischemia and other organ dysfunction [92]. Moreover, uremia may precipitate pericarditis through myocardial-depressant factors [59].

## 5. Conclusion

CRS type 3 represents a complicated clinical condition but the mechanisms whereby AKI causes cardiac dysfunction are not completely understood. Several pathways may be involved ncluding not only the activation of the immune and neuroendocrine systems, inflammatory mediators release, oxidative stress, and cellular apoptosis but also fluid overload, electrolyte, and acid-base imbalance and uremia. Continuous cellular and subcellular research in human and animal models will be able to elucidate the complex crosstalk in CRS type 3 as well as putative genetic modifications resulting from acute or chronic inflammatory states.

## Figures and Tables

**Table 1:** Cardiorenal syndrome (CRS) classification.

Acute cardiorenal syndrome	CRS type 1	Abrupt worsening of cardiac function leading to acute kidney injury, for example, acute heart failure; cardiac surgery; acute coronary syndrome; contrast-induced nephropathy

Chronic cardiorenal syndrome	CRS type 2	Chronic abnormalities of cardiac function causing chronic kidney disease, for example, Ischemic Heart Disease; Hypertension; chronic heart disease; chronic heart failure

Acute renocardiac syndrome	CRS type 3	Abrupt worsening of renal function leading to acute cardiac dysfunction Acute pulmonary edema in acute kidney injury; arrhythmia

Chronic renocardiac syndrome	CRS type 4	Chronic kidney disease leading to chronic cardiac dysfunction, for example, left ventricular hypertrophy; adverse cardiovascular events in chronic kidney disease

Secondary cardiorenal syndrome	CRS type 5	Systemic disorders causing both cardiac and renal dysfunction, for example, sepsis; systemic lupus erythematosus; diabetes mellitus

**Table 2:** Direct and indirect mechanisms of cardiorenal syndrome type 3.

Direct mechanisms	Systemic immune system	Innate
		Adaptive
	Inflammation, cytokines, and chemokines	TNF-*α*, IL-1, IL-6, ICAM-1
	Oxidative stress	RNS, ROS
	Apoptosis	Cardiac and renal cells
		Neutrophil infiltration
	SNS and RAAS	Norepinephrine activity, disturbance in myocardial calcium homeostasis, oxygen demand, cardiac myocite apoptosis

Indirect mechanisms	Fluid overload	Systemic edema, cardiac overload, hypertension, pulmonary edema, myocardial dysfunction
	Electrolyte imbalances	Hyperkalemia
		Hyperphosphatemia
		Hypophosphotemia
		Hypermagnesemia
	Acidemia	Alterations in protein structure and function
	Uremic toxins	Myocardial ischemia, pericarditis
